# Effect of Low Testosterone Levels on the Expression of Proliferator-Activated Receptor Alpha in Female Patients with Primary Biliary Cholangitis

**DOI:** 10.3390/cells12182273

**Published:** 2023-09-14

**Authors:** Agnieszka Kempińska-Podhorodecka, Joanna Abramczyk, Eliza Cielica, Bartosz Huła, Hanna Maciejowska, Jesus Banales, Piotr Milkiewicz, Małgorzata Milkiewicz

**Affiliations:** 1 Department of Medical Biology, Pomeranian Medical University, 70-111 Szczecin, Poland; agnieszka.kempinska.podhorodecka@pum.edu.pl (A.K.-P.); joanna.abramczyk@pum.edu.pl (J.A.); eliza.cielica@o2.pl (E.C.); bartoszhulapum@gmail.com (B.H.); h.maciejowska4@gmail.com (H.M.); malgorzata.milkiewicz@pum.edu.pl (M.M.); 2 Department of Liver and Gastrointestinal Diseases, Biodonostia Health Research Institute, Donostia University Hospital, University of the Basque Country (UPV/EHU), CIBERehd, Ikerbasque, 20014 San Sebastian, Spain; jesus.banales@biodonostia.org; 3 Department of Biochemistry and Genetics, School of Science, University of Navarra, 31009 Pamplona, Spain; 4 Liver and Internal Medicine Unit, Medical University of Warsaw, 02-097 Warsaw, Poland

**Keywords:** immune-mediated cholangitis, liver injury testosterone, peroxisome proliferator-activated receptor alpha

## Abstract

Sex-dependent patterns in chronic immune-mediated cholangiopathies, like primary biliary cholangitis (PBC) and primary sclerosing cholangitis (PSC), remain poorly understood. Peroxisome proliferator-activated receptor alpha (PPAR-α), expressed in immune cells, plays a key role in innate defence. In this study, the relationship between PPAR-α expression in peripheral blood mononuclear cells (PBMCs), serum androgen levels, IFNγ production, and sex-dependent tendencies during the development of PBC and PSC was investigated. We confirmed that normal cholangiocytes respond to PPAR-α and inhibit the lipopolysaccharide-induced expression of IL-6, IL-1b, and TNFα. Compared with PSC patients, PPAR-α was downregulated, while IFNγ was upregulated, in the PBMCs of PBC patients. When the analysis was conducted on females only, there was no difference in PPAR-α, but IFNγ was elevated in females with PBC compared with those with PSC. Serum testosterone concentrations in females with PBC were below the normal range (regardless of age) and correlated positively with PPAR-α and negatively with IFNγ. While PPAR-α has been reported to be a target of miR-155 and miR-21, no correlations with these microRNAs were observed in the PBMCs. However, a positive correlation between miR-21 and IFNγ was observed. Our results showed suppressed PPAR-α expression accompanied by reduced testosterone levels in women with PBC, which should elicit interest in the role of testosterone in PBC development.

## 1. Introduction

Two chronic cholangiopathies, primary biliary cholangitis (PBC) and primary sclerosing cholangitis (PSC), are characterised by immune-mediated liver injury. The hepatic influx of CD4+ and CD8+ T cells demonstrates cytotoxicity against bile duct cells and an imbalance between effector and regulatory T cells [1,2]. Furthermore, both diseases respond poorly to immunosuppression. Typical features of PBC include the presence of elevated plasma concentrations of specific anti-mitochondrial antibodies and female predominance. In contrast, PSC primarily affects men, and no disease-specific autoantibodies have been identified [1,2]. While numerous investigations into immune responses during cholestasis have been conducted, the mechanisms behind the sex differences observed in these liver diseases remain unknown.

It is commonly accepted that immune defences differ between males and females, and sexual dimorphism has been demonstrated in both mice and humans [3,4,5]. Compared with men, women are more likely to develop autoimmune diseases, which may be related to developing more robust type 1 T-helper (Th1) responses, as females’ naïve CD4+ T cells produce higher levels of type II interferon IFNγ than male T cells. IFN-γ, a cytokine produced by activated T cells and natural killer (NK) cells, maintains innate and adaptive immune responses. The production of Th1 and Th17 cytokines (e.g., IFNγ and IL-17A) is driven by the T cell production of two peroxisome proliferator-activated receptors (PPARs), PPAR-α and PPAR-γ. PPARs are transcription factors of a nuclear hormone receptor superfamily that, in adipose and liver tissues, are activated by various ligands and regulate whole-body energy homeostasis [6]. Furthermore, PPARs are expressed in immune cells and are crucial to enhancing innate host defences and inhibiting excessive inflammatory responses by promoting NF-κB inactivation [6,7,8]. PPAR-α stimulates an anti-inflammatory macrophage phenotype and affects the function of human T cells. PPAR-α expression is higher in CD4+ T cells from males than those from females, which is influenced by androgen levels [6,7,8]. Androgens increase PPAR-α and decrease PPAR-γ expression in human CD4+ T cells [7]. Similarly, the control of the hepatic expression of PPAR-α during inflammation is gender-dependent [9].

MicroRNA (miR) is the short product of a non-coding RNA gene that modulates gene expression by inhibiting the stability or translational efficiency of its target mRNA [10]. The distinct expression patterns of miRNAs have been described in specific T cell subsets, which likely reflect their proliferative history and differentiation stage j [11]. Several miRNAs, including miR-155, are involved in the development of Th1 and Th17 immune responses, whereas miR-21 is functionally involved in CD4 T cell activation and is altered upon T cell receptor stimulation. Recently, PPAR-α was reported to be a direct target of either miR-155 or miR-21 in biliary, hepatic, and inflammatory cells in a mouse model of alcohol-induced steatohepatitis or lupus alveolar haemorrhages and the development of hepatocellular carcinoma [12,13,14,15,16]. We previously showed that the experimental over-expression of both miR-155 and miR-21 suppresses PPAR-α levels in human hepatic cells (HepG2) but not in human cholangiocytes [17].

This study investigated a possible link between PPAR-α activity, androgen levels, IFNγ production, and sex-dependent tendencies during the development of autoimmune disorders in patients with PBC and PSC. Given that PPAR-α has been reported to be a target of miR-21 and miR-155 (and that these microRNAs are strongly upregulated in the livers of patients with PBC), we also analysed their expressions in peripheral blood mononuclear cells (PBMCs) and sought to determine their effect on PPAR-α expression.

## 2. Materials and Methods

### 2.1. Subjects

PBMCs from patients with PBC (n = 15), PSC (n = 33) and healthy individuals (n = 11) were isolated from blood samples taken from each study participant once in the morning between 8.00 a.m. and 9.00 a.m., between 2014 to 2016, and stored at −80 °C. Serum testosterone concentrations were analysed in female patients with PBC (n = 93) and PSC (n = 20). Patient demographic and clinical data are summarized in Table 1. The Ethics Committee of Pomeranian Medical University (BN-001/43/06) approved the study protocol, which was conducted according to the Declaration of Helsinki (6th revision, 2008). All patients provided written, informed consent to participate in the study.

### 2.2. Cell Culture

NHCs were scattered on a 6-well plate for cell adhesion to the vessel surface. Cells were cultured in media containing DMEM F12 with GlutaMax (Gibco, Thermofisher Scientific, Waltham, MA, USA) and Penicillin/Streptomycin (Biowest, Nuaille, France), Fetal Bovine Serum H.I. (ATCC 30-2025), MEM vitamin solution (Gibco, Thermofisher Scientific, Waltham, MA, USA), MEM Non-Essential AAs (Gibco, Thermofisher Scientific, Waltham, MA, USA), Lipid Mixture chemically defined (Sigma, Sigma-Aldrich, Milwaukee, WI, USA), Epidermal Growth Factor (Sigma, Sigma-Aldrich, Milwaukee, WI, USA), Soybean Trypsin Inhibitor (Gibco, Thermofisher Scientific, Waltham, MA, USA), Insulin Transferrin Selenium (Gibco, Thermofisher Scientific, Waltham, MA, USA), T3 (3,3′5-triiodo-L-thyronine), (Sigma, Sigma-Aldrich, Milwaukee, WI, USA), Dexamethasone (Sigma, Sigma-Aldrich, Milwaukee, WI, USA), Bovine Pituitary Extract (Gibco, Thermofisher Scientific, Waltham, WI, USA), and Forskolin (Sigma, Sigma-Aldrich, Milwaukee, WI, USA).

NHC cells were incubated with LPS (liposaccharide of *Escherichia coli*; 100 µM) for 24 h to initiate the inflammatory process or were pre-treated with fenofibrate (FB; 200 µM) for 2 h prior to LPS stimulation.

### 2.3. MicroRNA and mRNA Extraction and Quantification

Total RNA was isolated from patient PBMCs and NHCs using the RNeasy Mini Kit (Qiagen, Hilden, Germany) according to the manufacturer’s protocol. cDNA synthesis was carried out using the SuperScript IV-First-Strand cDNA Synthesis System Kit (Applied Biosystems, Thermo Fisher Scientific, Waltham, MA, USA) according to the manufacturer’s protocol. The transcripts of PPAR-α (Hs00947539_m1), IL-6 (Hs001741131_m1), IL-1b(Hs01555410_m1), TNFα(Hs00174128_m1, IL-17a (Hs00174383_m1), INF-γ (Hs00989291_m1), and 18S rRNA (Hs99999901_s1) were measured using the 7500 Fast Real-Time PCR System (Applied Biosystems, Waltham, MA, USA). Briefly, each assay comprised a 10 μL reaction mixture that contained 5 μL of TaqMan^®^ Gene Expression Master Mix (Applied Biosystems, Waltham, MA, USA), 1.5 μL of diluted cDNA template, and 0.5 μL of the probe/primer assay mix. Eukaryotic 18S ribosomal RNA served as an endogenous control.

For microRNA quantification, total RNA was isolated using the miRNeasy Serum/Plasma Advanced Kit (Qiagen), and cDNA was synthesised using the TaqMan Advanced miRNA cDNA Synthesis Kit (Applied Biosystems, Waltham, MA, USA) according to the manufacturer’s protocol. The levels of miR-155 (ID: 002623_mir) and miR-21 (ID: 477975_mir) along with miR-16 (ID: 477860_mir), which was used to correct for variations in RNA input/cDNA synthesis, were measured using TaqMan^®^ Advanced miRNA Assays (Applied Biosystems, Waltham, MA, USA).Each assay for miRNA expression comprised a 10 μL reaction mixture that contained 5 μL of TaqMan^®^ Fast Advanced Master Mix (2×) (Applied Biosystems), 2.5 μL of diluted cDNA, 0.5 μL of the TaqMan^®^ Advanced miRNA Assay (20×), and 2 μL of RNase-free water. Mean Ct values for all genes were quantified with the Sequence Detection Software, version 2.0.2 (Life Technologies, Thermo Fisher Scientific). The ΔCt method (ΔCt = Ct gene—Ct reference gene) was used to quantify target gene expression. Fold change expression levels were determined using the 2^−ΔΔCt^ formula.

### 2.4. ELISA Analyses

Serum testosterone concentrations were determined using a competitive Testosterone ELISA Kit (ab108666, Abcam, Cambridge, UK) according to the manufacturer’s protocol.

### 2.5. Statistics

Data are expressed as mean ± SEM for continuous variables and were analysed using the StatView 5 Software (SAS Institute, Cary, NC, USA). The variable distribution was tested for normality using the Shapiro–Wilks test. Differences between normally distributed variables were examined using one-way ANOVA with Fisher’s test with PLSD. The correlation coefficient was assessed using Z-tests. All in vitro experiments were repeated at least 3 times on separate occasions. The level of significance was set at *p* < 0.05 for all analyses.

## 3. Results

### 3.1. Fenofibrate Inhibits LPS-Induced Pro-Inflammatory Cytokines in Cholangiocytes

**Figure 1:**
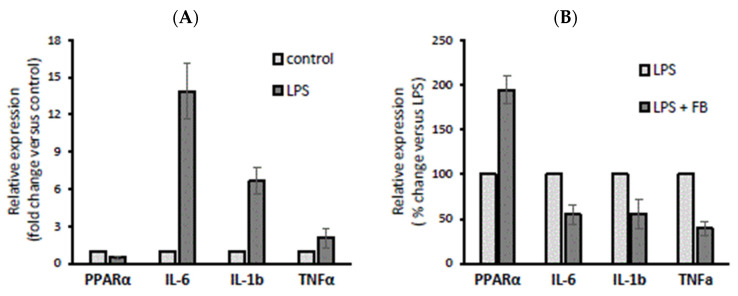
The effect of lipopolysaccharide (LPS) and fenofibrate (FB) stimulation on peroxisome proliferator-activated receptor α (PPAR-α), interleukin 6 (IL-6), interleukin-1 beta (1L-1b), and tumour necrosis factor-α (TNFα) expression in normal human cholangiocytes (NHCs). Incubation with LPS (100 µM) reduced PPAR-α and upregulated IL-6 and IL-1b after 24 h (**A**), whereas pre-treatment with FB (200 µM) for 2 h prior to LPS stimulation upregulated PPAR-α and suppressed IL-6, IL-1b, and TNFα (**B**). At least three independent experiments were conducted. Levels of gene expression were normalized to the endogenous reference 18S RNA. Bars indicate the mean ± SEM.

Recent studies have indicated that PPAR-α activation plays an important role in the function of innate and adaptive immune cells [6]. Cholangiocytes, which are primarily affected in cholestatic diseases, are now recognised as active players in immune pathogeneses, which induce immune-mediated ductular damage and release mediators regulating immune function [18]. Therefore, we evaluated the effect of the PPAR-α agonist fenofibrate (FB) on pro-inflammatory cytokines in normal human cholangiocytes (NHCs). In comparison with unstimulated NHCs, their incubation with LPS (100 µM) reduced PPAR-α by 53% (*p* = 0.006) and upregulated IL-6 and IL-1b after 24 h (*p* = 0.001 and *p* = 0.002, respectively; Figure 1A). Alternatively, pre-treatment with FB (200 µM) for 2h prior to LPS stimulation upregulated PPAR-α by 90% (*p* = 0.001 vs. LPS; Figure 1) and suppressed IL-6, IL-1b, and TNFα (by 46%, *p* = 0.006 vs. LPS; by 45%, *p* = 0.003 vs. LPS; and by 60%, *p* = 0.001 vs. LPS, respectively; Figure 1B).

### 3.2. The Expression of PPAR-α and IFNγ mRNA in Mononuclear Cells of PSC and PBC Patients

**Figure 2:**
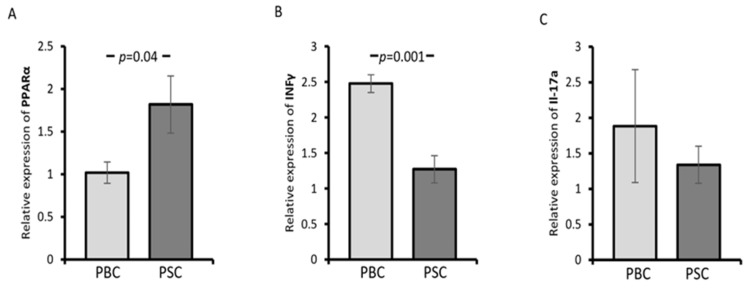
The expression of PPAR-α, interferon-gamma (IFNγ), and interleukin 17a (IL-17a) mRNA in the peripheral blood mononuclear cells (PBMCs) of PSC and PBC patients. Compared with PSC patients, the expression of PPAR-α was reduced in PBC patients (**A**), whereas IFNγ was upregulated (**B**). No difference was observed in IL-17a mRNA expression between PBC and PSC patients (**C**). Levels of gene expression are presented as fold changes and were normalised to the endogenous reference 18S RNA. Bars indicate the mean ± SEM.

Ductopenia and portal inflammation in PBC result from direct immune reactions focused on intrahepatic cholangiocytes. In PBMCs isolated from PBC patients, the expression of PPAR-α was reduced, whereas IFNγ was upregulated compared with PSC patients (*p* = 0.04 and *p* = 0.001, respectively; Figure 2A,B). No difference was observed between these groups regarding IL-17a mRNA expression (*p* = 0.47; Figure 2C).

**Figure 3:**
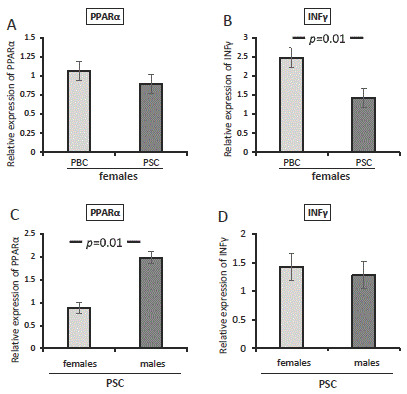
The expression of PPAR-α and IFNγ mRNA in patients with PBC or PSC. There was no difference between female PBC and PSC patients in their expression of PPAR-α (**A**). A higher expression of IFNγ was observed in females with PBC compared with those with PSC (**B**). In all PSC patients, females had lower levels of PPAR-α than males (**C**), but no difference in the relative expression of IFNγ was observed (**D**). Levels of gene expression are presented as fold changes and were normalised to the endogenous reference 18S RNA. Bars indicate the mean ± SEM.

Since a strong male predisposition has been observed in PSC patients, we conducted an additional analysis for females with PBC or PSC. When PPAR-α mRNA was evaluated, there was no difference between PBC and PSC females (*p* = 0.37; Figure 3A). However, a higher expression of IFNγ was observed in females with PBC compared with those with PSC (*p* = 0.01; Figure 3B). In contrast, when the analysis was made for the entire group of PSC patients, females had lower levels of PPAR-α compared with males (*p* = 0.012; Figure 3C), but no difference in the relative expression of IFNγ was observed (*p* = 0.68; Figure 3D).

### 3.3. The Serum Concentration of Testosterone in Female Patients with PBC and PSC

**Figure 4:**
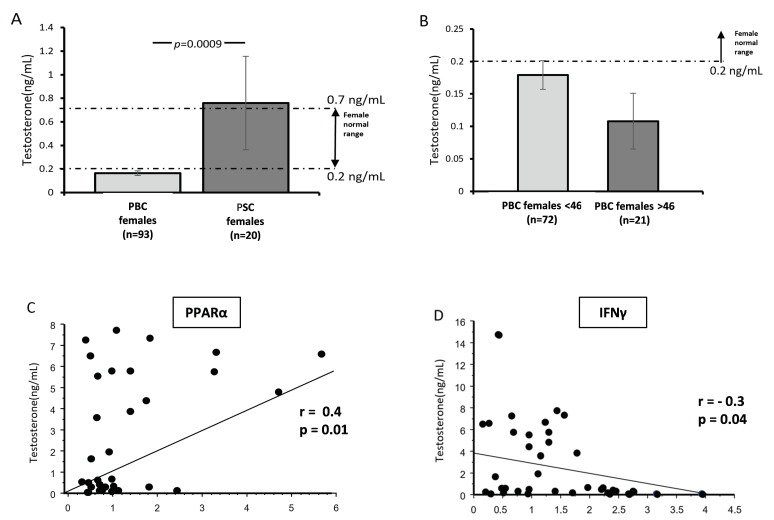
The serum concentration of testosterone in females with PBC and PSC. Bar charts show considerably lower concentrations of testosterone in the sera of female patients with PBC than in females with PSC (**A**). Testosterone levels were below the normal range in females with PBC, and there was no difference between younger (PBC < 46 years old) and older women (PBC > 46 years old). The dotted lines represent the normal range for females (**B**). The serum concentration of testosterone correlated positively with PPAR-α mRNA (**C**) and negatively with IFNγ mRNA (**D**). Dots illustrate each patient.

Given that androgens increase PPAR-α expression in T cells, we evaluated the serum concentration of testosterone in female subjects with PBC (n = 96) and PSC (n = 18). We observed considerably lower concentrations of testosterone in the sera of female patients with PBC than in those with PSC (*p* = 0.04; Figure 4A), which was below the normal range (i.e., less than 0.2 ng/mL). Moreover, we confirmed that this phenomenon was not related to age, as there was no difference in testosterone levels between younger (PBC < 46 years old) and older women (PBC > 46 years old) (*p* = 0.14; Figure 4B). The serum concentration of testosterone correlated positively with PPAR-α mRNA (r = 0.4; Z-value, 2.59; *p* = 0.009; Figure 4C) and negatively with IFNγ mRNA (r = −0.3; Z-value, 2.07; *p* = 0.04; Figure 4D). There was no correlation between the age at diagnosis, aspartate transferase (AST), alkaline phosphatase (ALP), fibrosis, bilirubin, or serum testosterone levels within the total patient group.

### 3.4. The Expression of miR-155 and miR-21 in PBMCs

**Figure 5:**
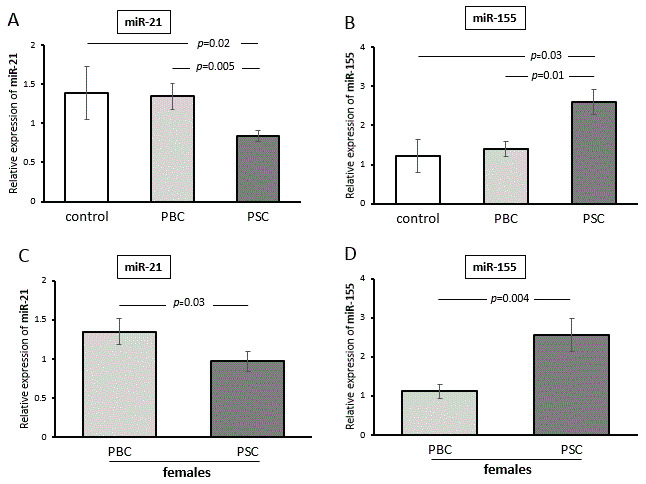
The expressions of miR-155 and miR-21 in PBMCs. The relative expression of miR-21 was lower in PSC patients compared with both controls and PBC patients (**A**). The difference between the diseases was similar when the analysis was made with only female patients (**B**). The expression of miR-155 was higher in PSC patients compared with PBC patients (**C**). The disease-specific variation was also maintained when only female patients were evaluated (**D**). Levels of miRNA expression were normalized to the endogenous reference miR-16. Bars indicate the mean ± SEM.

As some miRs, including miR-155 and miR-21, can inhibit PPAR-α gene activity, and the expressions of these miRs have been reported to be gender-dependent, we investigated the levels of these miRs in PBMCs. The relative expression of miR-21 was lower in PSC patients than in controls and PBC patients (*p* = 0.02 and *p* = 0.005, respectively; Figure 5A). The difference between the diseases was similar when the analysis was made only with female patients (PSC vs. PBC, *p* = 0.03; Figure 5B). In contrast, the expression of miR-155 was higher in PSC patients compared with PBC patients (PSC vs. PBC, *p* = 0.005; Figure 5C), and this disease-specific variation was also maintained when only female patients were evaluated (PSC vs. PBC, *p* = 0.004; Figure 5D).

## 4. Discussion

An acknowledgement of sex differences in immunity, especially in the context of autoimmune diseases, is increasingly being reported [3,4]. However, the role of sex hormones, particularly androgens, in the pathogenesis of PBC is scant and poorly understood. This study showed that PPAR-α is reduced in the PBMCs of PBC patients and associated with the induction of IFNγ. It is tempting to speculate that the low levels of testosterone we observed may mediate the development of PBC and be responsible for the inhibition of PPAR-α and a more robust Th1 response.

We observed a different expression of PPAR-α mRNA in PBMCs isolated from patients with chronic cholestatic liver diseases (i.e., PBC versus PSC)—conditions that are negatively affected by inflammation. The expression of this gene was substantially suppressed in PBC patients compared with PSC patients; to the best of our knowledge, this is a novel observation. Previously, PPAR-α inhibition was reported in the livers of PBC patients [17]. In contrast, the upregulation of this gene was observed in the PBMCs of patients with non-alcoholic fatty liver disease [19]. However, until now, PPAR-α expression has not been evaluated in the mononuclear cells of patients with cholestatic immune-mediated conditions, even though PPAR-α activation via FB is an FDA-approved adjunct therapy for ursodeoxycholic acid-refractory PBC patients. The beneficial effects of FB, a PPAR-α agonist, may contribute to various actions, such as regulating bile acid synthesis and bile excretory functions and maintaining cholesterol and lipid homeostasis. Of importance, PPAR-α also plays a role in the inhibition of excessive inflammatory responses either through negative interference with the pro-inflammatory transcription factors NF-κB and c-jun or the reinforcement of macrophage polarization toward an anti-inflammatory phenotype [20].

Given our finding that the lower expression of PPAR-α in PBC vs. PSC may be biased by female dominance in PBC, we increased the size of the group of PSC patients by 16 female patients (female PSC group). When the analysis was made with only female patients, the disease-related difference in PPAR-α expression disappeared. However, when the analysis was conducted with only PSC patients, the females produced less PPAR-α than males. This suggests that PPAR-α production in chronic cholestatic disease is gender-dependent.

The observed suppression of PPAR-α in the PBMCs of PBC patients was accompanied by the upregulation of the Th1 cytokine IFNγ. While our study did not address the molecular mechanisms underlying IFNγ repression via PPAR-α, a previous study showed that it involves the recruitment of nuclear receptor co-repressor 1 to the IFNγ gene and reducing histone acetylation [7]. Moreover, it has been suggested that human T cells exhibit a sex difference in the production of IFNγ, which is driven by PPAR-α [7]. PPAR-α is expressed at higher levels in the T cells of males and appears to control Th1 cytokine production in male mice [8]. Furthermore, Th cells from female mice produce more IFNγ than T cells from male mice [7]. Moreover, experimentally induced autoimmune encephalomyelitis in castrated mice leads to the higher production of IFNγ compared with sham mice, suggesting that androgen shifts cytokine production [7]. FB reduced IFNγ but only in wild-type and not in PPAR-α^−/−^ T cells or males. This confirms a sex-specific role for PPAR-α in the negative regulation of T-cell IFNγ production. Similarly, in response to anti-CD3 and anti-CD28 stimulation, females exhibit a higher production of IFNγ than their male counterparts [7].

A large body of evidence suggests that androgen levels shape gender dimorphism in cytokine production [21,22,23]. Exposure to testosterone in vivo results in (i) a reduction in NK cell activity and leukotriene biosynthesis [24]; (ii) the inhibition of TNF and inducible nitric oxide synthase (iNOS) synthesis caused by macrophages; and (iii) the enhancement of IL-10 and transforming growth factor beta (TGFβ) production [25]. In immune cells, PPAR-α is at the juncture of gender and immune regulations, as androgen-induced PPAR-α suppresses IFNγ and TNF production in T cells [7].

This sexual dimorphism led us to investigate whether the lower expression of PPAR-α observed in female patients correlated with testosterone serum levels. We noticed a significantly lower circulating level of testosterone in PBC female patients vs. females with PSC. In PBC female patients, testosterone levels were below the normal range (0.2–2.0 ng/mL) both in younger, premenopausal, and older postmenopausal women. Previously, a decreased testosterone level in male and female patients was associated with autoimmune diseases, including systemic lupus erythematosus, rheumatoid arthritis, Sjogre’s syndrome, and multiple sclerosis [26,27,28,29]. Our results from PBC patients are in line with a previous report by Floreani et al. [30], which suggested that alterations in sex hormone profiles are secondary to hepatic dysfunction. However, in our study, we observed a significant difference between the two cholestatic diseases (which were characterised by hepatic dysfunction), and the testosterone levels did not correlate with either ALP or fibrosis.

The serum concentration of testosterone in women, although more than 10-fold lower than in men, is essential for immune regulation [31]. Androgens directly or indirectly affect T cell phenotypes and functions, and androgen receptors are in the cells of the innate and adaptive immune systems, thymocytes, and cholangiocytes [22,32,33,34]. Androgens, including testosterone, suppress pro-inflammatory responses via (1) the inhibition of TNF, iNOS, and nitric oxide synthesis caused by macrophages; (2) the augmentation of anti-inflammatory responses caused by IL-10 and TGFβ stimulation; and (3) the induction of regulatory T-cell (CD4+FOXP3+) frequencies via the stimulation of the Treg master transcription factor, FOXP3 [35]. A protective effect following testosterone treatment was observed in a mouse model of multiple sclerosis and lupus [36,37]. The treatment of female mice with testosterone inhibits the secretion of IFNγ by NK T cells [38]. Similarly, testosterone suppressed liver inflammation in a model of experimental cholangitis with a high female predominance [39]. In humans, pilot studies have shown a reduction in disease symptoms after testosterone treatment in females with autoimmune diseases such as systemic lupus erythematosus, rheumatoid arthritis, multiple sclerosis, and Sjogre’s syndrome [26,27,28,29]. However, those studies used small patient cohorts and lacked meticulous clinical characteristics.

Regarding the miR-dependent modulation of PPAR-α expression, we observed no correlation with miR21 or miR-155 levels in PBMCs. However, we did notice a positive correlation between IFNγ and miR-21 expression. MiR-21 is a key switch that influences the magnitude of inflammation and promotes an anti-inflammatory, immunosuppressive environment. It acts as a negative modulator of toll-like receptor 4 and TNF-a, which results in the elevated production of IL-10, the reduced secretion of IL-6, and the suppression of excessive inflammation [40,41]. MiR-21 also regulates the balance between Th1 and Th2 responses [42,43]. It has been proposed that miR-21 regulates adaptive immune responses via the attenuation of the IL-12/IFNγ pathway [42]. Conversely, IFN induces miR-21 expression, and this effect is cell-context-dependent [44]. This is in line with our observations showing the suppressed expression of IFNγ was accompanied by the hindered expression of miR-21 in PBMCs.

An obvious limitation of this study is a lack of experiments focused on the effect of various testosterone concentrations on PPAR-α expression in cultured normal human cholangiocytes (NHCs), immortalized human cholangiocytes (H69), and PBC-like cholangiocytes with an induced overexpression of miR-506 (H69-miR506). This work is planned for the near future. Also, the results would be even more convincing if we had a higher number of PBMC samples available to us.

## 5. Conclusions

In conclusion, our findings demonstrating the suppressed expression of PPAR-α accompanied by reduced serum testosterone levels in female PBC patients should elicit interest in the role of testosterone in the development of PBC.

## Figures and Tables

**Table 1:** Demographic and clinical characteristics of the study participants.

	PBMC				Serum		
	Control (n = 11)	PBC (n = 15)	PSC (n = 19)	PSC Female (n = 14)	PBC < 46 (n = 72)	PBC > 46 (n = 21)	PSC (n = 20)
Gender (male/female)	1/10	1/14	15/4	0/14	0/72	0/21	0/20
Age (years)	36 ± 6	55 ± 7	34 ± 11	38 ± 11	45 ± 5	65 ± 13	38 ± 9
Bilirubin (mg/dL, norma l < 1.1)	WNR	1.1 ± 0.9	2.3 ± 1.8	1.3 ± 0.4	2.1 ± 3.1	1.5 ± 1.1	4.2 ± 3.0
ALP (IU/L, normal 30–120)	WNR	235 ± 142	387 ± 234	261 ± 157	263 ± 237	392 ± 354	290 ± 154
AST (IU/L, normal 5–35)	WNR	53 ± 33	71 ± 34	65 ± 50	74 ± 77	80 ± 56	74 ± 49
ALT (IU/L, normal < 40)	WNR	57 ± 41	75 ± 39	83 ± 50	111 ± 161	101 ± 96	88 ± 48
Albumin (g/dL, normal 3.8–4.2)	WNR	3.8 ± 0.4	4.1 ± 0.5	4.1 ± 1.3	4.2 ± 0.5	3.9 ± 0.4	4.2 ± 0.6

ALP—alkaline phosphatase; ALT—alanine aminotransferase; AST—aspartate aminotransferase; WNR—within normal range.

## Data Availability

Not applicable.
